# Doença Cardiovascular e Fatores de Risco em Mulheres na América Latina e no Caribe

**DOI:** 10.36660/abc.20250655

**Published:** 2026-08-03

**Authors:** Claudio T. Mesquita, Amalia Peix, Diana I. Rodríguez, Karen A. Dueñas-Criado, Claudia Gutiérrez-Villamil, Adriana Puente, Teresa Massardo, Isabel Berrocal, Andrea Astesiano, Roberto N. Agüero, Ryenne Bañolas, Enrique Hiplan, Mayra Sánchez, Ana Ma. Barreda, Vanessa V. Gómez, Cynthia Fernández, Silvia Portillo, Yariela Herrera, Aurelio Mendoza, Miguel Kapitan, Carlos Castellanos, Enrique Estrada-Lobato, Diana Paez, Giovane L. Azevedo

**Affiliations:** 1 Hospital Universitário Antonio Pedro-Ebeserh UFF Niterói RJ Brasil Hospital Universitário Antonio Pedro-Ebeserh (UFF), Niterói, RJ – Brasil; 2 Instituto de Cardiología y Cirugía Cardiovascular La Habana Cuba Instituto de Cardiología y Cirugía Cardiovascular, La Habana – Cuba; 3 Nuclear Medicine and Diagnostic Imaging Section Division of Human Health International Atomic Energy Agency Vienna Áustria Nuclear Medicine and Diagnostic Imaging Section, Division of Human Health, International Atomic Energy Agency, Vienna – Áustria; 4 Fundación Cardioinfantil Instituto de Cardiología Bogotá Colômbia Fundación Cardioinfantil-Instituto de Cardiología, Bogotá – Colômbia; 5 Centro Médico Nacional ‘20 de Noviembre’ Ciudad de México México Centro Médico Nacional ‘20 de Noviembre’, ISSSTE, Ciudad de México – México; 6 Universidad de Chile Hospital Clínico Santiago Chile Universidad de Chile - Hospital Clínico, Santiago – Chile; 7 Hospital San Juan de Dios Caja Costarricense de Seguro Social San José Costa Rica Hospital San Juan de Dios - Caja Costarricense de Seguro Social, San José – Costa Rica; 8 Fundación Escuela Medicina Nuclear Mendoza Argentina Fundación Escuela Medicina Nuclear (FUESMEN), Mendoza – Argentina; 9 Fundación Centro Diagnóstico Nuclear Buenos Aires Argentina Fundación Centro Diagnóstico Nuclear (FCDN), Buenos Aires – Argentina; 10 Hospital Base Valdivia Chile Hospital Base, Valdivia – Chile; 11 Hospital de Especialidades ‘Carlos Andrade Marín’ Quito Equador Hospital de Especialidades ‘Carlos Andrade Marín’, Quito – Equador; 12 Hospital Oncológico del ISSS San Salvador El Salvador Hospital Oncológico del ISSS, San Salvador – El Salvador; 13 Hospital María de Especialidades Pediátricas Tegucigalpa Honduras Hospital María de Especialidades Pediátricas, Tegucigalpa – Honduras; 14 Instituto Nacional Cardiopulmonar Tegucigalpa Honduras Instituto Nacional Cardiopulmonar, Tegucigalpa – Honduras; 15 Hospital Santo Tomás Ciudad de Panamá Panamá Hospital Santo Tomás, Ciudad de Panamá – Panamá; 16 Instituto Nacional Cardiovascular Lima Perú Instituto Nacional Cardiovascular INCOR, Lima – Perú; 17 Hospital Italiano Montevideo Uruguai Hospital Italiano, Montevideo – Uruguai; 18 Hospital Dr. Carlos Arvelo Caracas Venezuela Hospital Dr. Carlos Arvelo, Caracas – Venezuela

**Keywords:** Doenças Cardiovasculares, América Latina, Mulheres, Imageamento Multimodal

## Abstract

**Fundamento:**

A doença cardiovascular (DCV) permanece como a principal causa de morte entre mulheres na América Latina e no Caribe (ALC), sendo a doença isquêmica do coração o principal componente. Apesar desse importante impacto, os programas de saúde da mulher na região tradicionalmente priorizaram a prevenção do câncer, frequentemente negligenciando a DCV.

**Objetivo:**

Analisar a carga de DCV e a distribuição dos fatores de risco cardiovascular entre mulheres na ALC, destacando diferenças relacionadas ao sexo e disparidades regionais.

**Métodos:**

Este estudo analisou dados epidemiológicos sobre mortalidade por DCV e fatores de risco cardiovascular nos países da ALC, incluindo fatores comportamentais, metabólicos e específicos do sexo feminino, além de comparações com países selecionados fora da região.

**Resultados:**

As mulheres na ALC apresentaram maior prevalência de sedentarismo, dislipidemia, glicemia de jejum elevada e sobrepeso/obesidade, enquanto o tabagismo foi mais frequente entre os homens. Fatores de risco específicos do sexo feminino – como gravidez na adolescência, parto prematuro, diabetes gestacional e distúrbios hipertensivos da gestação – foram altamente prevalentes, porém sub-reconhecidos, sendo amplamente influenciados por determinantes socioeconômicos. Em 2021, a COVID-19 superou temporariamente a doença isquêmica do coração e o acidente vascular cerebral como principal causa de morte nas Américas (176,7 óbitos por 100.000 habitantes), evidenciando a vulnerabilidade de mulheres com risco cardiometabólico prévio. A mortalidade prematura por DCV variou amplamente: as maiores probabilidades de morte entre 30 e 70 anos foram observadas no Haiti (21,9%) e na Guiana (15,2%), associadas, respectivamente, à hipertensão mal controlada e ao colesterol elevado. Em contraste, o Canadá apresentou a menor mortalidade prematura (1,7%), refletindo sistemas de saúde mais robustos e menor prevalência de fatores de risco metabólicos.

**Conclusão:**

A DCV continua sendo a principal causa de mortalidade entre mulheres na ALC, caracterizada por importantes disparidades regionais e elevada carga de fatores de risco tradicionais e específicos do sexo feminino. Esses achados reforçam a necessidade urgente de estratégias específicas ao sexo e sensíveis ao contexto, integrando a prevenção cardiovascular às políticas abrangentes de saúde da mulher, superando abordagens centradas exclusivamente no câncer.

## Graphical abstract

**Figura Central:**
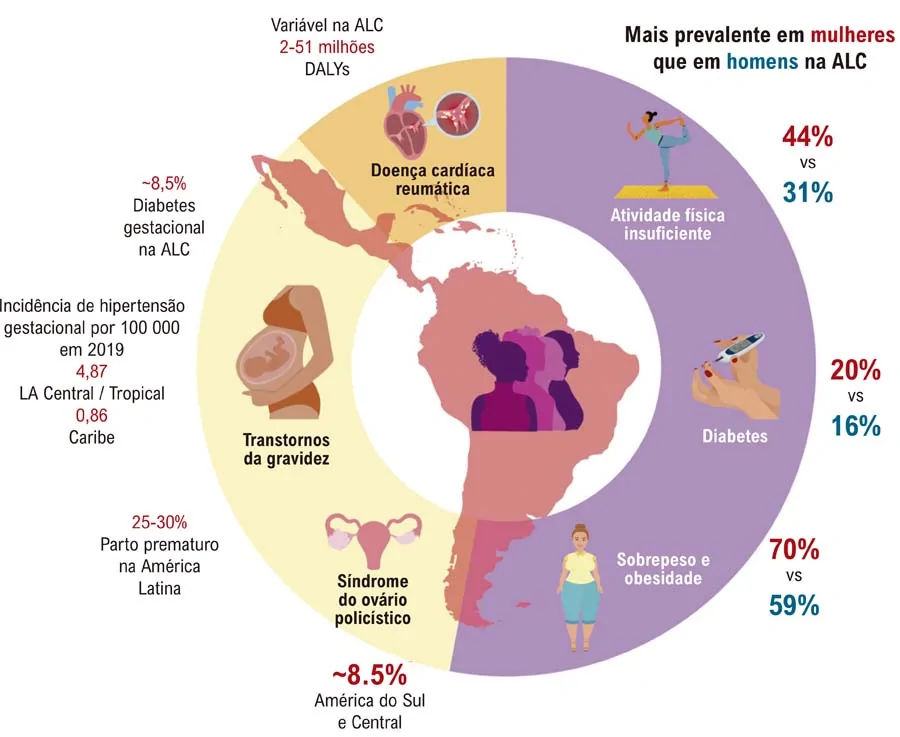
: Doença Cardiovascular e Fatores de Risco em Mulheres na América Latina e no Caribe

## Introdução

Em 2019, as 10 principais causas de morte foram responsáveis por 55% da mortalidade global, com a doença isquêmica do coração (DIC) permanecendo como a principal causa, responsável por 16% de todos os óbitos. Notavelmente, a DIC apresentou o aumento mais acentuado nas últimas décadas, passando de pouco mais de 2 milhões de mortes em 2000 para 8,9 milhões em 2019.^1^

Na América Latina e no Caribe (ALC), as doenças cardiovasculares (DCV) são a principal causa de morte, com taxas de mortalidade específicas por causa de 184 por 100 000 entre homens adultos e 108 por 100 000 entre mulheres adultas – substancialmente superiores às médias relatadas nos países da Organização para a Cooperação e Desenvolvimento Econômico (OCDE), de 104 e 53 por 100 000, respectivamente.^2^ Além disso, entre mulheres mais jovens, a mortalidade por infarto agudo do miocárdio tem sido relatada como maior do que entre homens em diversas regiões.^3-6^ Desde então, novos dados epidemiológicos surgiram, e a pandemia de COVID-19 remodelou substancialmente as tendências de mortalidade em todo o mundo, incluindo na ALC, onde temporariamente superou as DCV como principal causa de morte. Essas mudanças reforçam a necessidade de análises atualizadas e novas revisões para reavaliar a carga crescente das DCV, particularmente entre mulheres.

Embora as DCV sejam a principal causa de mortalidade entre mulheres na ALC, a conscientização sobre doenças cardiovasculares nesse grupo permanece baixa em toda a região.^7-12^ Alguns estudos mostram que mulheres da ALC têm maior probabilidade do que os homens de apresentar indicadores aumentados de adiposidade e três ou mais fatores de risco cardiovascular.^13^ De modo geral, a maioria dos casos de DCV e das mortes prematuras na ALC poderia ser evitada por meio do controle eficaz de fatores de risco metabólicos e do tabagismo, que continuam sendo os determinantes mais comuns e mais potentes desses desfechos na região.^14,15^

As mulheres continuam sub-representadas em setores-chave, como economia, educação e pesquisa científica, perpetuando disparidades estruturais. A igualdade de gênero não é apenas um direito humano fundamental, mas também um fator essencial para a saúde global, a equidade e a sustentabilidade. Crises recentes de saúde, incluindo a pandemia de COVID-19, juntamente com os efeitos contínuos das mudanças climáticas, influenciaram ainda mais a mortalidade cardiovascular e a prevalência de fatores de risco, ampliando as lacunas de gênero.^16^ Enfrentar esses desafios exige a geração de dados confiáveis e atualizados, capazes de orientar políticas de saúde alinhadas aos Objetivos de Desenvolvimento Sustentável – particularmente o ODS 3: Saúde e Bem-Estar – por meio do monitoramento dos fatores de risco e de seu impacto nas comunidades.^12,17,18^

Para obter uma visão atualizada e compreender os mecanismos que contribuem para o aumento das DCV em mulheres, a Agência Internacional de Energia Atômica (AIEA) iniciou um projeto regional com o objetivo de melhorar a conscientização sobre DCV por meio da identificação de lacunas em educação e treinamento. A AIEA oferece cooperação técnica a 31 países da ALC, auxiliando os Estados-Membros a enfrentar prioridades essenciais de desenvolvimento em áreas como saúde, meio ambiente e aplicações industriais.

Este artigo tem como objetivo descrever a situação atual das DCV na região das Américas, como ponto de partida para a implementação de iniciativas no âmbito do projeto ARCAL 6086, apoiado pela AIEA: “Integração de técnicas de medicina nuclear em uma abordagem multimodal em cardiologia para diagnóstico precoce e estratificação de risco de doenças cardiovasculares em mulheres latino-americanas”. Este estudo fornece uma análise regional e específica por sexo sobre DCV em mulheres na ALC. Ao integrar dados de mortalidade entre países, risco de DCV prematura (idades de 30 a 70 anos), fatores cardiometabólicos tradicionais, determinantes específicos do ciclo de vida feminino e o impacto da COVID-19 em um único arcabouço, buscamos ir além das estimativas descritivas de carga de doença e oferecer uma perspectiva sensível ao contexto e orientada a políticas, adaptada às realidades dos sistemas de saúde da ALC.

## Métodos

Este estudo foi delineado como uma análise ecológica descritiva baseada exclusivamente em dados secundários obtidos no portal ENLACE da Organização Pan-Americana da Saúde (OPAS) para doenças não transmissíveis e saúde mental.^18^ O objetivo foi caracterizar a carga de DCV e os fatores de risco associados em mulheres nos países da ALC, dentro do contexto mais amplo da região das Américas.

Analisamos os dados mais recentes disponíveis na OPAS, incluindo taxas de mortalidade e anos de vida ajustados por incapacidade (DALYs, do inglês *disability-adjusted life years*) referentes a 2021. Além disso, examinamos a prevalência dos principais fatores de risco ateroscleróticos utilizando dados de 2022 para diabetes, atividade física insuficiente, sobrepeso e obesidade; dados de 2019 para prevalência de hipertensão; e dados de 2018 para taxas de tabagismo e níveis médios de colesterol total.

O estudo apresenta indicadores epidemiológicos padronizados conforme fornecidos pela OPAS, incluindo incidência, prevalência, taxas de mortalidade e DALYs. Os dados foram analisados para todos os grupos etários e estratificados por sexo (feminino, masculino e ambos os sexos combinados). A ênfase foi colocada nos padrões de mortalidade específicos das mulheres e na mortalidade prematura por DCV (idades de 30 a 70 anos), quando disponível.

Os 36 países incluídos na análise foram: Antígua e Barbuda, Argentina, Bahamas, Barbados, Belize, Bolívia (Estado Plurinacional da), Brasil, Canadá, Chile, Colômbia, Costa Rica, Cuba, Dominica, República Dominicana, Equador, El Salvador, Granada, Guatemala, Guiana, Haiti, Honduras, Jamaica, México, Nicarágua, Panamá, Paraguai, Peru, Porto Rico, São Cristóvão e Nevis, Santa Lúcia, São Vicente e Granadinas, Suriname, Trinidad e Tobago, Estados Unidos da América, Uruguai e (República Bolivariana da) Venezuela. Embora os Estados Unidos e o Canadá não sejam países da ALC, foram incluídos para fins comparativos, em conformidade com a classificação regional das Américas utilizada pela OPAS.

O ENLACE funciona como um repositório regional abrangente que integra dados primários provenientes dos sistemas nacionais de estatísticas vitais oficialmente reportados pelos Estados-Membros, inquéritos populacionais de saúde e estimativas globais harmonizadas da Organização Mundial da Saúde (OMS) e do estudo *Global Burden of Disease* (GBD). Esses procedimentos de integração são desenvolvidos pela OPAS para aprimorar a padronização dos dados e a comparabilidade entre países.

A análise concentrou-se na descrição e comparação de indicadores epidemiológicos padronizados entre países e entre os sexos. Todos os resultados são apresentados como estimativas descritivas derivadas dos dados reportados pela OPAS, e as interpretações são limitadas a padrões populacionais, sem implicar associações causais.

Este estudo utilizou dados secundários, agregados e de acesso público, provenientes da plataforma ENLACE da OPAS; portanto, não houve necessidade de aprovação por comitê de ética nem de consentimento informado. Todos os dados estão disponíveis no portal da OPAS, e nenhum conjunto de dados primários ou código analítico foi gerado.

## Resultados

### Carga global de doenças cardiovasculares nos países da ALC

As doenças cardiovasculares – principalmente a doença isquêmica do coração (DIC) e as doenças cerebrovasculares, sobretudo o acidente vascular cerebral – continuam sendo as principais causas de mortalidade e importantes contribuintes para os DALYs. Em 2021, a COVID-19 foi a principal causa de carga de doença na Região das Américas para ambos os sexos.^19^

De acordo com as estimativas mais recentes da OPAS para 2021, as principais causas de morte nas Américas foram: COVID-19 (176,7 óbitos por 100.000 habitantes), DIC (114,0 por 100.000) e acidente vascular cerebral (48,4 por 100.000), seguidas pela doença de Alzheimer e outras demências (38,1 por 100.000) e pela doença pulmonar obstrutiva crônica (DPOC) (34,6 por 100.000). Quando estratificadas por sexo, a segunda e a terceira causas de mortalidade permaneceram DIC e AVC; entretanto, a quarta causa diferiu: entre homens, violência interpessoal; entre mulheres, doença de Alzheimer (ver Figura 1).^18^

**Figura 1:**
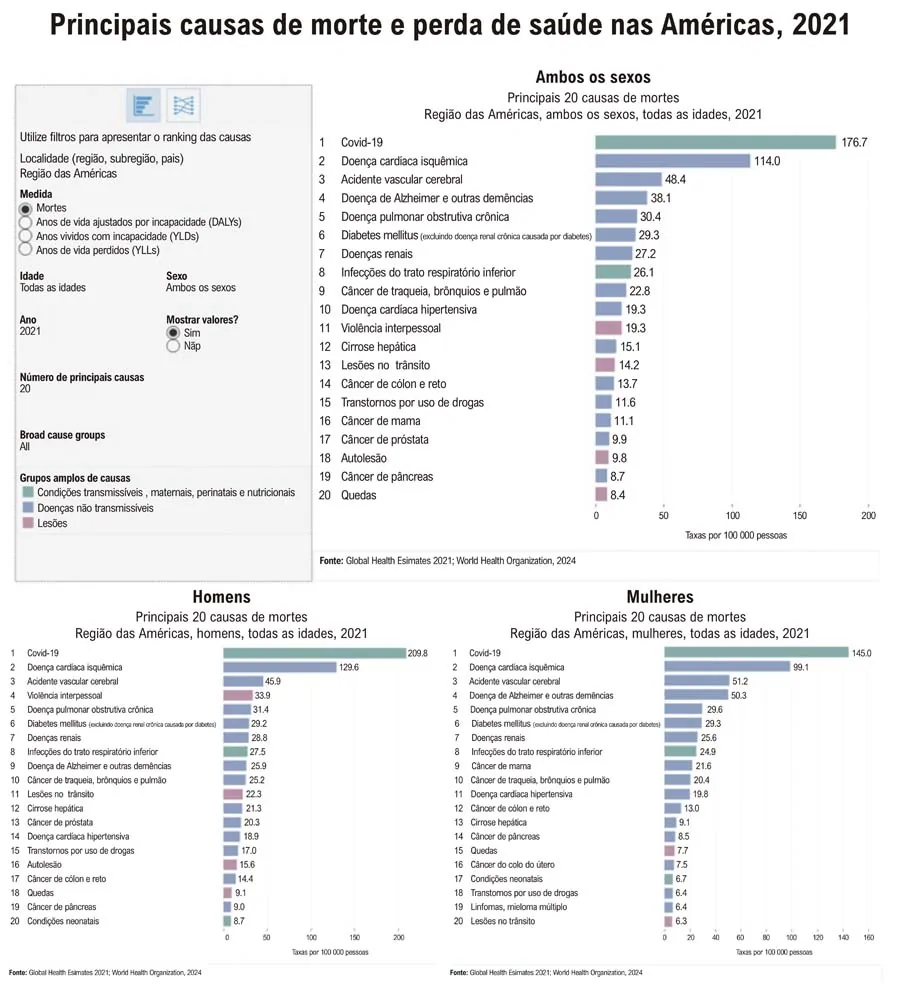
– Principais causas de morte e perda de saúde nas Américas, 2021 (ambos os sexos, homens e mulheres). Adaptado da Pan American Health Organization.47

A mortalidade prematura por DCV – medida como a probabilidade incondicional de morrer entre as idades exatas de 30 e 69 anos – é um dos indicadores monitorados pela Organização Mundial da Saúde. Quanto maior essa probabilidade, maior o impacto das DCV na mortalidade de um país. A desagregação desses dados por sexo ajuda a esclarecer disparidades na carga de DCV entre homens e mulheres.

A Figura 2 mostra a probabilidade de morrer por DCV entre as idades de 30 e 69 anos, estratificada por sexo. O país com a menor mortalidade prematura por DCV entre mulheres na região é o Canadá (1,7%). Essa probabilidade é mais de 12 vezes maior no Haiti (21,9%). Entre os homens, Peru (4,4%) e Guatemala (4,4%) apresentaram as menores taxas de mortalidade prematura por DCV. No extremo oposto, as maiores probabilidades também foram observadas no Haiti (20,1%) e na Guiana (17,7%).

**Figura 2:**
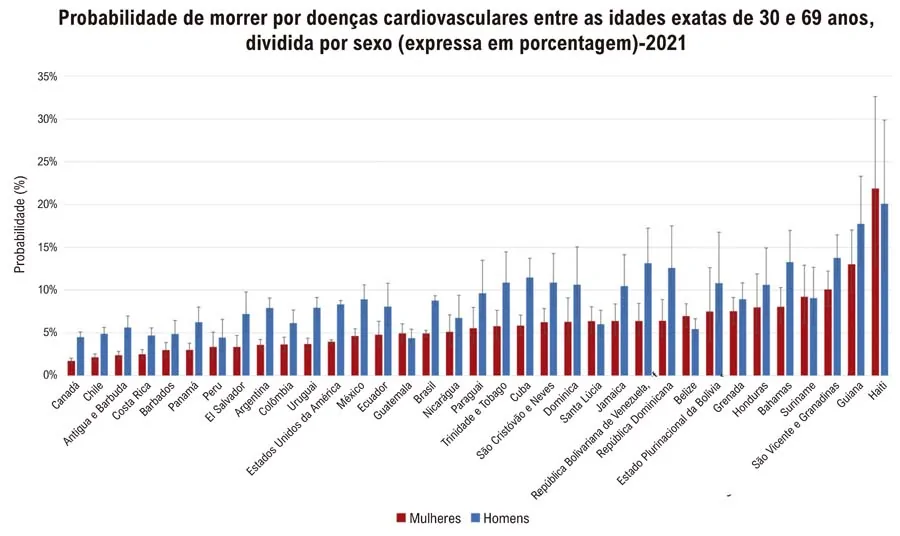
– Probabilidade de morrer por doenças cardiovasculares entre as idades exatas de 30 e 69 anos, dividida por sexo (expressa em porcentagem); adaptado da Pan American Health Organization.48

A probabilidade de morrer por DCV entre 30 e 69 anos é maior entre mulheres apenas no Haiti (1,8 pontos percentuais acima dos homens). Em todos os demais países das Américas, o risco de mortalidade prematura por DCV é maior entre homens do que entre mulheres. Suriname e Guiana apresentaram as maiores diferenças entre os sexos: 6,7 pontos percentuais mais alta entre homens na Venezuela e 6,2 pontos percentuais mais alta na República Dominicana.

### Fatores de risco ateroscleróticos

Os fatores de risco cardiovascular para mulheres incluídos nesta revisão clínica são aqueles tradicionalmente conhecidos, tais como: hipertensão arterial, diabetes mellitus, tabagismo, obesidade e sedentarismo. Além disso, fatores específicos do sexo feminino têm sido identificados, relacionados ao histórico e às condições próprias das mulheres.^20-26^ A análise a seguir avalia quais países apresentam maior prevalência desses fatores e os possíveis determinantes subjacentes. Todos os países da região das Américas foram incluídos para facilitar a comparação.

#### Níveis de colesterol

A média padronizada por idade do colesterol total nos países das Américas em 2018 variou de 164 mg/dL em Barbados a 185 mg/dL no Uruguai entre os homens, e de aproximadamente 173 mg/dL em Barbados a mais de 192 mg/dL na Guiana entre as mulheres (Figura 3a). Em quase todos os países, as mulheres apresentaram níveis de colesterol mais elevados do que os homens, com exceção do Chile.

**Figura 3:**
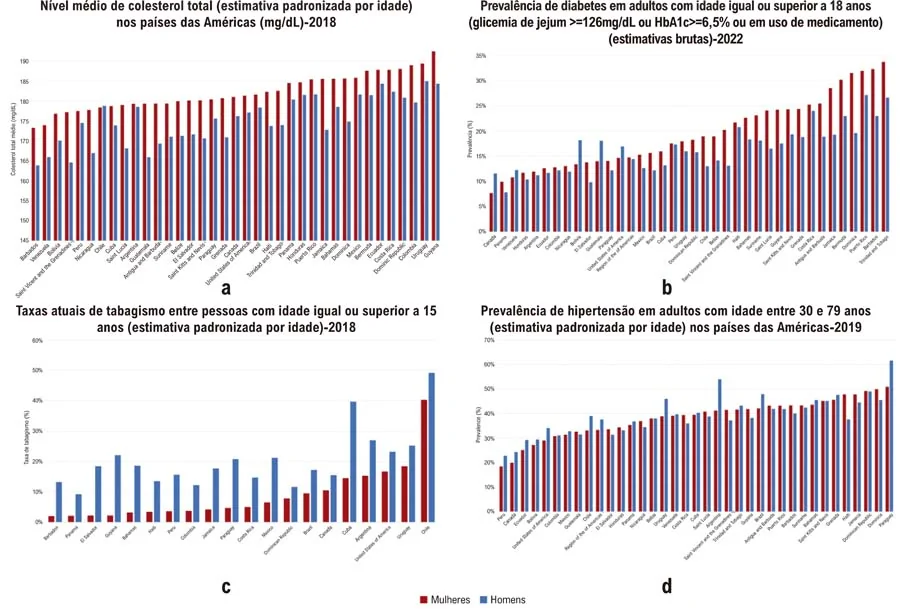
– Principais fatores de risco cardiovascular nos países das Américas: nível médio de colesterol total (a); prevalência de diabetes em adultos (b); taxas de tabagismo atual entre pessoas com 15 anos ou mais (estimativa padronizada por idade) (c); prevalência de hipertensão entre adultos de 30 a 69 anos (estimativa padronizada por idade) (d). Adaptado da Pan American Health Organization49,50 e World Health Organization.51

#### Prevalência de diabetes em adultos

A prevalência de diabetes em adultos (glicemia de jejum ≥126 mg/dL, HbA1c ≥6,5% ou uso atual de medicação) entre indivíduos com 18 anos ou mais é apresentada na Figura 3b. A prevalência variou amplamente entre os países, indo de cerca de 7,7% no Canadá a mais de 33,7% em Trinidad e Tobago entre as mulheres. Entre os homens, a menor prevalência foi observada no Panamá (7,8%) e a maior em Porto Rico (27,2%). Em média, a prevalência nos países da ALC foi notavelmente elevada — afetando uma em cada cinco mulheres (20%) e quase um em cada seis homens (16%) — conforme ilustrado na Figura Central.

A prevalência de diabetes em adultos foi geralmente maior entre mulheres do que entre homens na maioria dos países, com exceções nos Estados Unidos, Canadá, Venezuela, Bolívia e Guatemala. As maiores diferenças entre os sexos foram observadas em Dominica, Jamaica e Barbados, onde as mulheres apresentaram prevalência marcadamente superior à dos homens.

#### Tabagismo

Em 2018, a prevalência de tabagismo foi consistentemente maior entre homens do que entre mulheres em todos os países das Américas. Entre as mulheres, a prevalência foi inferior a 20% na maioria dos países, exceto no Chile, onde 32,7% das mulheres relataram fumar (Figura 3c). Os países com as maiores prevalências de tabagismo feminino foram Chile (32,7%), Estados Unidos (17,3%), Argentina (16,6%) e Uruguai (16,0%). Na outra extremidade, as menores prevalências entre mulheres foram observadas em Barbados (2%), Panamá (2,1%) e El Salvador (2,2%).

Entre os homens, a prevalência de tabagismo foi substancialmente maior, ultrapassando 30% no Chile e em Cuba. As maiores diferenças entre os sexos foram observadas em Cuba, Guiana e El Salvador, sendo Cuba o país com a maior disparidade — o tabagismo foi 25,2 pontos percentuais mais prevalente entre homens. Em contraste, a República Dominicana apresentou a menor diferença entre os sexos, com apenas 3,8 pontos percentuais.

#### Hipertensão arterial

Foi identificada uma prevalência heterogênea de hipertensão entre adultos de 30 a 79 anos (Figura 3d). A hipertensão foi definida como pressão arterial sistólica ≥130 mmHg, pressão arterial diastólica ≥80 mmHg ou uso autorreferido de medicação anti-hipertensiva.

Os países no quintil mais alto de prevalência de HBP foram Paraguai (56,4%), República Dominicana (49,1%), Dominica (47,7%), Argentina (47,5%), Granada (46,6%), Jamaica (46,3%), São Cristóvão e Nevis (45,1%) e Brasil (45,0%). Em comparação, Guatemala (32,2%), México (32,1%), Estados Unidos (31,6%), Colômbia (31,0%), Bolívia (28,3%), Equador (27,2%), Canadá (22,1%) e Peru (20,7%) compuseram o quintil mais baixo.

Interessantemente, a prevalência de hipertensão foi relativamente menor entre mulheres na maioria dos países. Argentina e Paraguai apresentaram as maiores diferenças entre os sexos: 12,8 pontos percentuais mais alta entre homens na Argentina (54,0% vs. 41,2%) e 10,7 pontos percentuais mais alta entre homens no Paraguai (61,6% vs. 50,9%). No sentido oposto, o Haiti apresentou a maior prevalência de hipertensão entre mulheres em comparação aos homens, com diferença de 10,2 pontos percentuais (37,6% vs. 47,8%). O país com a maior prevalência de hipertensão entre mulheres foi o Paraguai (50,9%).

#### Atividade física insuficiente

Diferentemente da hipertensão arterial, a atividade física insuficiente é mais prevalente entre mulheres do que entre homens em toda a ALC. A proporção de mulheres sedentárias é altamente heterogênea na região (Figura 4a). Os países no quintil mais alto de inatividade física entre mulheres foram Cuba (72%), Panamá (66,9%), Costa Rica (57,8%), Suriname (55,1%) e Trinidad e Tobago (54,2%). Em contraste, as menores prevalências foram observadas em Santa Lúcia (24,0%), Bolívia (26,6%), Equador (27,8%), México (30,5%) e Haiti (35,8%).

O sedentarismo foi consistentemente mais comum entre mulheres. As maiores diferenças entre os sexos foram observadas em Cuba, Guiana e Bahamas, cada uma apresentando mais de 20 pontos percentuais de maior prevalência de inatividade entre mulheres em comparação aos homens. Em média, nos países da ALC, 44% das mulheres e 31% dos homens relataram atividade física insuficiente (Figura Central).

#### Sobrepeso e obesidade

A prevalência de sobrepeso e obesidade (índice de massa corporal ≥25 Kg/m^2^) nos países das Américas, por sexo, é apresentada na Figura 4b. Entre as mulheres, a prevalência foi muito elevada, variando de 38,4% no Haiti a 81,9% em São Cristóvão e Nevis. Entre os homens, a menor prevalência foi observada no Haiti (21,0%), enquanto a maior ocorreu no Chile (78,5%). De modo geral, o sobrepeso e a obesidade foram mais comuns entre mulheres do que entre homens na maioria dos países, com exceções no Canadá, Estados Unidos, Argentina e Uruguai.

**Figura 4:**
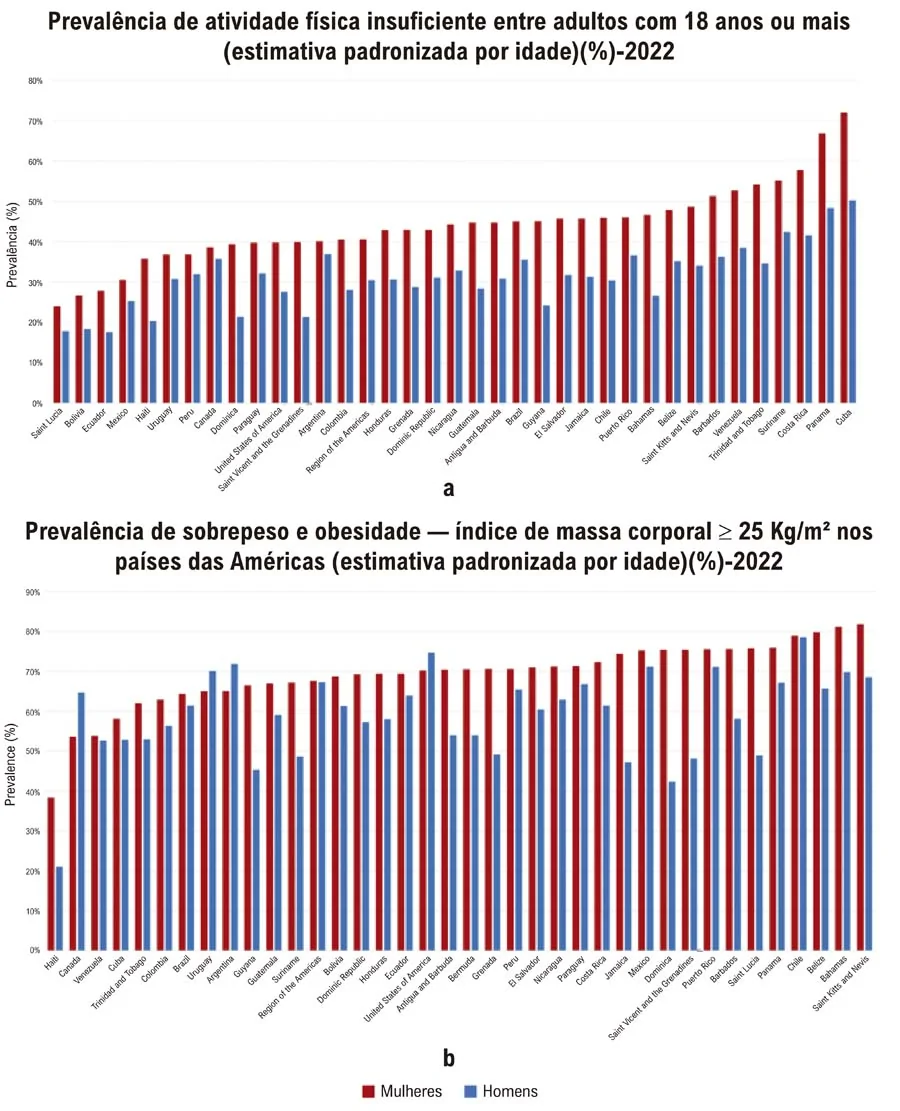
– Prevalência de atividade física insuficiente entre adultos com 18 anos ou mais (estimativa padronizada por idade) (a) e prevalência de sobrepeso e obesidade — índice de massa corporal ≥ 25 Kg/m2 (b) nos países das Américas. Adaptado da Pan American Health Organization.52

As maiores diferenças entre mulheres e homens foram encontradas em Dominica, Jamaica, São Vicente e Granadinas e Santa Lúcia, onde a prevalência entre mulheres excedeu a dos homens em mais de 25 pontos percentuais. Em média, nos países da ALC, 70% das mulheres e 59% dos homens apresentavam sobrepeso ou obesidade (Figura Central).

## Discussão

Este estudo oferece uma análise regional da carga de DCV em mulheres da ALC, destacando que, apesar do impacto excepcional da pandemia de COVID-19, as DCV continuam sendo a principal causa de morte na região. Nossos achados, derivados do banco de dados ENLACE, demonstram uma prevalência preocupante de fatores de risco cardiometabólicos tradicionais que afetam desproporcionalmente as mulheres da ALC em comparação aos homens. Embora esses dados evidenciem a magnitude do problema em nível populacional, a transição desses padrões epidemiológicos para o risco clínico individual é fortemente influenciada por determinantes específicos do sexo. Fatores biológicos e desfechos adversos na gestação atuam como importantes multiplicadores de risco, ainda sub-reconhecidos nas agendas de saúde regionais.

Nesse contexto, é essencial discutir como condições específicas do sexo feminino interagem com o perfil tradicional de risco observado em nossa análise. É amplamente reconhecido que fatores genéticos e ambientais podem modificar significativamente o risco cardiovascular em mulheres. Alguns são diretamente influenciados pelos hormônios sexuais, especialmente os estrogênios, que exercem efeitos cardiovasculares protetores durante os anos reprodutivos; outros são moldados por mecanismos genéticos e epigenéticos, incluindo distúrbios relacionados à gestação, que impactam o risco ao longo de todo o curso de vida. No contexto da ALC, vários desses determinantes têm sido estudados especificamente, reforçando a necessidade de considerar a saúde cardiovascular das mulheres sob uma perspectiva de curso de vida, abrangendo as fases reprodutiva, climatérica e pós-menopausa.^27-29^

A síndrome dos ovários policísticos afeta de 4% a 20% das mulheres em idade reprodutiva. Sua prevalência relatada em mulheres latino-americanas é variável: estima-se 6,6% (IC 95%: 2,3–10,9%) em mulheres mexicanas e cerca de 8,5% na América Central e do Sul (ver Ilustração Central). O diagnóstico é estabelecido pela presença de dois dos seguintes critérios: 1) hiperandrogenismo, 2) ovários policísticos e 3) disfunção ovariana. A síndrome está associada a um risco significativamente maior (5 a 10 vezes) de fatores de risco cardiometabólicos, como obesidade, síndrome metabólica, diabetes mellitus tipo 2, hipertensão arterial e dislipidemia, que aumentam o risco de DCV em comparação com a população geral (OR 1,5; IC 95%: 1–1,2). Por isso, o cuidado com o estilo de vida é fundamental no controle do sobrepeso, da obesidade e na prevenção do desenvolvimento de diabetes mellitus.^30,31^

Os distúrbios relacionados à gestação são cada vez mais reconhecidos como importantes fatores de risco cardiovasculares específicos do sexo feminino. Condições como os distúrbios hipertensivos da gravidez (por exemplo, pré-eclâmpsia, eclâmpsia), diabetes gestacional e parto prematuro aumentam significativamente o risco de morbimortalidade cardiovascular tanto durante a gestação quanto ao longo da vida. Na ALC, essas complicações são particularmente preocupantes devido às desigualdades estruturais: muitas mulheres enfrentam barreiras de acesso aos serviços de saúde, oportunidades limitadas de rastreamento e baixa conscientização entre profissionais de saúde sobre suas implicações cardiovasculares de longo prazo. Enfrentar essas lacunas exige maior capacitação dos profissionais de saúde, inclusão sistemática do histórico gestacional na avaliação de risco cardiovascular e integração dessas variáveis em prontuários eletrônicos e diretrizes nacionais de prevenção. Ampliar o acesso ao acompanhamento pós-parto e a programas de prevenção direcionados em países de baixa e média renda também será essencial para reduzir a carga de DCV a longo prazo entre mulheres.^32^

A prevalência global relatada de diabetes gestacional varia amplamente, de 1% a 28%, e é de aproximadamente 8,5% (IC 95%: 3,9%–14,7%) na ALC (ver Ilustração Central). Especificamente, a Federação Internacional de Diabetes relata uma prevalência de 20,7% na América do Norte e Caribe, 15,8% na América Central e do Sul e de 8,1%, 10,6% e 9,8% na Argentina, Brasil e Chile, respectivamente, com prevalências ainda mais elevadas em Cuba (24,6%) e México (13,3%).^33,34^

Mulheres com diabetes gestacional apresentam maior incidência de eventos cardiovasculares nos anos após o parto. Seus filhos também enfrentam riscos aumentados de obesidade, pré-diabetes e diabetes tipo 2 desde a infância e ao longo de toda a vida (20%). Mulheres com diabetes gestacional têm probabilidade substancialmente maior de desenvolver diabetes tipo 2 e síndrome metabólica (20–70%), além de um risco duas vezes maior de eventos cardiovasculares maiores nos anos após o parto, em comparação com mulheres sem a condição. Portanto, após o parto, toda mulher diagnosticada com diabetes gestacional deve receber orientação sobre mudanças no estilo de vida e sobre os riscos de longo prazo de desenvolver essas condições, incluindo o risco elevado de eventos cardiovasculares mais de 10 anos após o parto.^33^

Os distúrbios hipertensivos da gravidez estão entre as principais causas de morbidade e mortalidade materna e fetal. Eles englobam várias condições com prognósticos e abordagens terapêuticas distintas, incluindo hipertensão gestacional, pré-eclâmpsia, eclâmpsia e pré-eclâmpsia sobreposta à hipertensão crônica.^35^ Há um aumento exponencial no risco de desenvolver DCV após uma gestação complicada por pré-eclâmpsia, e esse risco cresce com a idade: uma mulher de 20 anos, com risco basal de 0,6%, vê esse risco aumentar para 1,2%, enquanto em uma mulher de 40 anos o risco sobe de 5,6% para 11,2%.^36^ A incidência global de hipertensão na gravidez aumentou de 16,30 milhões (IC 95%: 13,56–19,42 milhões) em 1990 para 18,08 milhões (IC 95%: 15,26–21,11 milhões) em 2019, representando um aumento de 10,92%.^37^ Em 2019, a incidência por 100.000 mulheres variou substancialmente na ALC. Conforme mostrado na Ilustração Central, as maiores taxas ocorreram nas sub-regiões Central e Tropical (4,87; IC 95%: 4,27–5,54) e as menores no Caribe (0,86; IC 95%: 0,69–1,04).^37,38^ O risco elevado de DCV persiste por 10 a 20 anos após a gestação, ressaltando a importância do monitoramento da pressão arterial e da adoção precoce de mudanças no estilo de vida após uma gravidez hipertensiva.^35^

O parto prematuro, definido como aquele que ocorre antes de 37 semanas de gestação, também está associado ao aumento da mortalidade cardiovascular. Os mecanismos que relacionam o parto prematuro à DCV envolvem fatores de risco cardiovasculares pré-existentes, inflamação sistêmica e subsequente disfunção vascular. A incidência relatada varia globalmente, com as menores taxas (<5%) na Europa e as maiores na África (18%). Aproximadamente 60% dos partos prematuros no mundo ocorrem na África Subsaariana e no Sul da Ásia. A ALC ocupa uma posição intermediária, com variações importantes entre sub-regiões (25–30%, ver Ilustração Central). Estima-se que entre 5,8% e 7,1% das gestantes no mundo tenham tido pelo menos um parto prematuro. As tecnologias de reprodução assistida também estão associadas a maiores riscos de parto prematuro e de parto no início do termo, e seu uso tem aumentado na América Latina, especialmente entre mulheres com gestação única.^39^

A doença cardíaca reumática causa 0,7 mortes por 100 000 habitantes na região das Américas. A doença valvar cardíaca é um importante contribuinte para a perda de capacidade funcional, qualidade de vida e longevidade da população. A doença reumática permanece, de longe, a manifestação mais comum da doença primária em todo o mundo e afeta aproximadamente 40,5 milhões de pessoas, com uma incidência anual de 2,8 milhões, sendo responsável por 306.000 mortes globais em 2019, com idade mediana de 28,7 anos em países de baixa e média renda.^40^ A epidemiologia desse distúrbio foi analisada com foco na distribuição geográfica e nas diferenças entre os sexos nas Américas.

Em relação à contribuição para a mortalidade cardiovascular total na região, os países com as maiores taxas entre mulheres em 2019 foram Haiti, Bolívia e Santa Lúcia. As mulheres apresentaram 50,9 milhões de DALYs por ano no Brasil, Costa Rica, Nicarágua e Santa Lúcia, com níveis mais baixos na Venezuela, Colômbia, Uruguai e Chile (2,0 milhões de DALYs por ano) (Figura Central). Entre os homens, observaram-se 9,2 milhões de DALYs na Costa Rica, Nicarágua, Santa Lúcia, Granada e Brasil, com níveis mais baixos na Venezuela, Colômbia, Chile e Uruguai (1,4; 1,5; 2,6 e 2,7 milhões, respectivamente). Existe uma variabilidade substancial dentro da região, tanto nacional quanto subnacional, nos DALYs atribuídos à doença cardíaca reumática — com valores significativamente mais elevados no Brasil, Costa Rica e Nicarágua em comparação com o restante da ALC.^40-42^

Nossa análise das DCV em mulheres na ALC destaca várias reflexões importantes. Primeiro, as DCV continuam altamente prevalentes, com a DIC entre as principais causas de mortalidade em mulheres em toda a região. Segundo, os fatores de risco cardiovascular tradicionais apresentam marcada heterogeneidade entre os sexos. Terceiro, os fatores de risco específicos do sexo feminino são ao mesmo tempo altamente prevalentes e frequentemente negligenciados. Condições como gravidez na adolescência, parto prematuro, diabetes gestacional e distúrbios hipertensivos da gravidez são fortemente influenciadas por determinantes socioeconômicos e permanecem sub-reconhecidas, apesar de sua contribuição bem estabelecida para o risco cardiovascular ao longo da vida. Em conjunto, esses achados reforçam a necessidade urgente de estratégias adaptadas para promover a saúde cardiovascular das mulheres na região, integrando determinantes biológicos, culturais e sociais.

Ao examinar os fatores de risco convencionais, a maioria dos países da ALC apresenta taxas mais elevadas de colesterol, hiperglicemia, atividade física insuficiente e sobrepeso/obesidade entre mulheres, enquanto o tabagismo permanece o único fator consistentemente mais prevalente entre homens. Esse desequilíbrio é preocupante, considerando que os programas de saúde da mulher na região historicamente enfatizaram a prevenção de cânceres (por exemplo, mama, colo do útero, ovário), dedicando menos atenção à prevenção de DCV — embora as DCVs sejam a principal causa de morte entre mulheres fora dos anos da pandemia de COVID-19. De fato, em 2021, a COVID-19 emergiu como a principal causa de mortalidade nas Américas (176,7 mortes por 100.000 habitantes), superando a DIC (114,0/100.000) e o AVC (48,4/100.000). Embora esse aumento represente uma circunstância excepcional, ele complicou ainda mais as prioridades de prevenção e sobrecarregou os sistemas de saúde, evidenciando a vulnerabilidade de mulheres com fatores de risco cardiometabólicos pré-existentes.

O estudo *Global Use of Strategies to Open Occluded Coronary Arteries in Acute Coronary Syndromes* (GUSTO IIb), publicado há duas décadas, já havia documentado diferenças entre os sexos nos fatores de risco cardiovascular e na apresentação das síndromes coronarianas agudas: as mulheres eram mais velhas que os homens e apresentavam hipertensão e diabetes com maior frequência, além de níveis mais elevados de colesterol e maior prevalência de histórico de angina, insuficiência cardíaca e doença cerebrovascular.^20^ Achados semelhantes têm sido consistentemente relatados, reforçando a necessidade de incluir adequadamente as mulheres em estudos clínicos e evitar a extrapolação de dados de DCV obtidos em homens para mulheres.^20^

A pesquisa da *American Heart Association* de 2019 mostrou uma queda na conscientização das mulheres sobre as DCV como sua principal causa de morte, passando de 65% em 2009 para 44% em 2019. As quedas mais acentuadas ocorreram entre mulheres jovens, hispânicas e negras, com um número crescente identificando incorretamente o câncer como a principal causa de morte. Esses achados ressaltam a necessidade urgente de renovar campanhas de educação e conscientização voltadas especificamente para a saúde cardiovascular das mulheres.^23^ Em alguns países da ALC, pesquisas semelhantes foram conduzidas, revelando que menos de 20% das entrevistadas reconhecem as DCV como a principal causa de morte.^24,25^

Quando desagregados por sexo, os óbitos globais anuais e os DALYs entre mulheres foram aproximadamente 13% menores do que entre homens; no entanto, as mulheres passaram cerca de 26% mais anos vivendo com incapacidade (YLDs, do inglês *years living with disability*).^21^ As diretrizes atuais de saúde da mulher tradicionalmente enfatizam a prevenção dos cânceres de mama, colo do útero e ovário, enquanto dedicam relativamente menos atenção às DCVs – a principal causa de morte entre mulheres, exceto durante a pandemia de COVID-19. As estratégias de cuidado preventivo frequentemente priorizam a saúde reprodutiva e o rastreamento de câncer, mas as evidências mostram que as mulheres enfrentam uma carga desproporcionalmente alta de fatores de risco cardiometabólicos, incluindo obesidade, hipertensão, diabetes e dislipidemia, que aumentam significativamente o risco cardiovascular ao longo da vida. Apesar disso, o rastreamento e o aconselhamento voltados à saúde cardiovascular permanecem subutilizados em comparação com intervenções focadas em câncer. Esse desequilíbrio precisa ser corrigido, pois tanto as DCVs quanto o câncer exigem estratégias preventivas dedicadas devido à sua elevada carga e fatores de risco sobrepostos.^22^ Em alinhamento com os Objetivos de Desenvolvimento Sustentável das Nações Unidas, a meta para 2030 é reduzir em um terço a mortalidade prematura por doenças crônicas não transmissíveis por meio da prevenção.

Os dados de mortalidade prematura reforçam ainda mais o impacto desigual das DCVs entre os países. Entre as mulheres, a probabilidade de morrer por DCV entre 30 e 70 anos foi mais alta no Haiti (21,9%) e na Guiana (15,2%). No Haiti, a hipertensão se destaca como o principal fator de risco (47,8%), enquanto as taxas de diabetes e tabagismo são relativamente baixas, sugerindo que deficiências no diagnóstico, tratamento ou manejo da hipertensão podem estar impulsionando a elevada mortalidade prematura no país. Já a Guiana apresentou alguns dos níveis mais altos de colesterol entre mulheres na região, juntamente com prevalências acima da média de outros fatores de risco cardiometabólicos, ampliando ainda mais a carga geral de risco.

No extremo oposto do espectro, o Canadá apresentou a menor mortalidade prematura por DCV entre mulheres (1,7%). Esse perfil favorável provavelmente reflete as vantagens de um país de alta renda, com infraestrutura de saúde mais robusta, melhor acesso ao cuidado preventivo e manejo mais eficaz dos fatores de risco. De fato, as prevalências de hiperglicemia, hipertensão, sedentarismo e sobrepeso/obesidade no Canadá estavam todas abaixo das médias regionais. Curiosamente, a prevalência de tabagismo entre as mulheres canadenses (10,5%) era cerca de três vezes maior do que no Haiti (3,4%), mas a mortalidade geral permaneceu substancialmente menor. Esse achado reforça a noção de que o agrupamento e a coexistência de múltiplos fatores de risco – e não exposições isoladas – desempenham um papel decisivo na carga de doença. Compreender essas interações é fundamental para desenvolver estratégias de prevenção mais eficazes e específicas ao contexto na ALC.

Embora nossos achados estejam alinhados com padrões previamente descritos – como a predominância da DIC e a elevada carga de fatores de risco metabólicos – nossa análise amplia referências anteriores ao focar especificamente na mortalidade prematura por DCV em mulheres (30–70 anos) e ao destacar a marcada heterogeneidade entre países da ALC.^27,32,43,44^ Também integramos fatores cardiometabólicos tradicionais com determinantes específicos do curso de vida feminino, incluindo gravidez na adolescência e distúrbios hipertensivos da gravidez, dentro de um arcabouço unificado. Além disso, situar nossos resultados no contexto da mortalidade pós-COVID-19 evidencia como a vulnerabilidade cardiometabólica subjacente ampliou as disparidades regionais, oferecendo uma interpretação mais abrangente e relevante para políticas de risco cardiovascular em mulheres.

Nossos achados ressaltam a necessidade de estratégias cardiovasculares coordenadas e específicas por sexo na ALC. As autoridades de saúde pública devem ampliar as agendas de saúde da mulher para além da prevenção de câncer, incorporando uma avaliação sistemática do risco cardiovascular ao longo do curso de vida, com foco especial no controle da hipertensão, rastreamento do diabetes, manejo de lipídios e combate à inatividade física. O acompanhamento estruturado de mulheres com desfechos adversos na gestação – como distúrbios hipertensivos da gravidez e diabetes gestacional – deve ser incorporado aos fluxos de cuidado da atenção primária. Os sistemas de saúde também precisam otimizar os processos de diagnóstico e tratamento da DIC em mulheres, garantir acesso equitativo às terapias recomendadas por diretrizes e fortalecer o encaminhamento para reabilitação cardíaca.

Dada a marcada heterogeneidade na mortalidade prematura por DCV entre mulheres nos diferentes países, as intervenções devem priorizar medidas escaláveis e custo-efetivas – especialmente o controle da pressão arterial – ao mesmo tempo em que implementam indicadores mensuráveis, incluindo taxas de rastreamento desagregadas por sexo, controle de fatores de risco e mortalidade prematura (30–70 anos), para monitorar o progresso ao longo do tempo.

Achados recentes de estudos de 2024 e 2025 sobre doenças cardíacas em mulheres na América Latina indicam que disparidades socioeconômicas, urbanização e determinantes sociais da saúde são importantes impulsionadores de risco e desfechos, com mulheres rurais enfrentando maiores barreiras de acesso ao cuidado e taxas mais elevadas de mortalidade.^43,45,46^ Há uma necessidade crítica de intervenções adaptadas que considerem as variações específicas de cada país em infraestrutura de saúde, acesso e estabilidade política.

Reconhecemos que o uso de diferentes anos de referência para distintos fatores de risco cardiovascular pode introduzir heterogeneidade temporal e potencialmente afetar a comparabilidade direta entre os indicadores. Portanto, os achados devem ser interpretados à luz dessa característica inerente aos bancos de dados internacionais de acesso aberto.

## Conclusão

Em resumo, as DCVs continuam sendo a principal causa de morte entre mulheres na ALC, impulsionadas por uma interação complexa entre fatores de risco tradicionais e específicos do sexo feminino, muitos dos quais são sub-reconhecidos e insuficientemente abordados nas agendas atuais de saúde. As marcantes disparidades entre os países destacam a influência do contexto socioeconômico, do acesso aos serviços de saúde e do agrupamento de fatores de risco nos desfechos. Esses achados reforçam a necessidade urgente de estratégias sensíveis ao contexto e específicas por sexo para aprimorar a prevenção, a detecção precoce e o manejo das DCV em mulheres, bem como uma integração mais robusta da saúde cardiovascular nos programas já existentes de saúde da mulher em toda a região.
