# Supplementary material for: Doença Cardiovascular e Fatores de Risco em Mulheres na América Latina e no Caribe
Source: Arq Bras Cardiol. 2026 Aug 3;123(7):e20250655. [Article in Portuguese] doi: 10.36660/abc.20250655 (PMC13668458; doi:10.36660/abc.20250655)
Supplement: Translation

# Translation: Cardiovascular Disease and Risk Factors in Women in Latin America and the Caribbean

**DOI:** 10.36660/abc.20250655i

**Authors:** Claudio T. Mesquita, Amalia Peix, Diana I. Rodríguez, Karen A. Dueñas-Criado, Claudia Gutiérrez-Villamil, Adriana Puente, Teresa Massardo, Isabel Berrocal, Andrea Astesiano, Roberto N. Agüero, Ryenne Bañolas, Enrique Hiplan, Mayra Sánchez, Ana Ma. Barreda, Vanessa V. Gómez, Cynthia Fernández, Silvia Portillo, Yariela Herrera, Aurelio Mendoza, Miguel Kapitan, Carlos Castellanos, Enrique Estrada-Lobato, Diana Paez, Giovane L. Azevedo Junior

**Affiliations:** 1 Brazil Hospital Universitário Antonio Pedro-Ebeserh (UFF), Niterói, RJ – Brazil; 2 Cuba Instituto de Cardiología y Cirugía Cardiovascular, La Habana – Cuba; 3 Austria Nuclear Medicine and Diagnostic Imaging Section, Division of Human Health, International Atomic Energy Agency, Vienna – Austria; 4 Colombia Fundación Cardioinfantil-Instituto de Cardiología, Bogotá – Colombia; 5 Mexico Centro Médico Nacional ‘20 de Noviembre’, ISSSTE, Ciudad de México – Mexico; 6 Chile Universidad de Chile - Hospital Clínico, Santiago – Chile; 7 Costa Rica Hospital San Juan de Dios - Caja Costarricense de Seguro Social, San José – Costa Rica; 8 Argentina Fundación Escuela Medicina Nuclear (FUESMEN), Mendoza – Argentina; 9 Argentina Fundación Centro Diagnóstico Nuclear (FCDN), Buenos Aires – Argentina; 10 Chile Hospital Base, Valdivia – Chile; 11 Ecuador Hospital de Especialidades ‘Carlos Andrade Marín’, Quito – Ecuador; 12 El Salvador Hospital Oncológico del ISSS, San Salvador – El Salvador; 13 Honduras Hospital María de Especialidades Pediátricas, Tegucigalpa – Honduras; 14 Honduras Instituto Nacional Cardiopulmonar, Tegucigalpa – Honduras; 15 Panama Hospital Santo Tomás, Ciudad de Panamá – Panama; 16 Peru Instituto Nacional Cardiovascular INCOR, Lima – Peru; 17 Uruguay Hospital Italiano, Montevideo – Uruguay; 18 Venezuela Hospital Dr. Carlos Arvelo, Caracas – Venezuela

**Keywords:** Cardiovascular Diseases, Latin America, Women, Multimodal Imaging

## Abstract

**Background:**

Cardiovascular disease (CVD) remains the leading cause of death among women in Latin America and the Caribbean (LAC), with ischemic heart disease as the primary contributor. Despite this burden, women’s health programs in the region have traditionally prioritized cancer prevention, often neglecting CVD.

**Objective:**

To analyze the burden of CVD and the distribution of cardiovascular risk factors among women in LAC, highlighting sex-related differences and regional disparities.

**Methods:**

This study analyzed epidemiological data on CVD mortality and cardiovascular risk factors across LAC countries, including behavioral, metabolic, and female-specific risk factors, and conducted comparisons with selected countries outside the region.

**Results:**

Women in LAC showed a higher prevalence of sedentary lifestyle, dyslipidemia, elevated fasting blood glucose, and overweight/obesity, whereas smoking was more frequent among men. Female-specific risk factors – such as teenage pregnancy, preterm birth, gestational diabetes, and hypertensive disorders of pregnancy – were highly prevalent yet under-recognized, largely shaped by socioeconomic determinants. In 2021, COVID-19 temporarily surpassed ischemic heart disease and stroke as the leading cause of death in the Americas (176.7 deaths per 100,000 population), highlighting the vulnerability of women with underlying cardiometabolic risk. Premature CVD mortality varied widely, with the highest female probabilities of death between ages 30 and 70 observed in Haiti (21.9%) and Guyana (15.2%), associated with poorly controlled hypertension and elevated cholesterol, respectively. In contrast, Canada showed the lowest premature mortality (1.7%), reflecting stronger healthcare systems and lower prevalence of metabolic risk factors.

**Conclusion:**

CVD remains the leading cause of mortality among women in LAC, marked by substantial regional disparities and a significant burden of both traditional and female-specific risk factors. These findings highlight the urgent need for sex-specific, context-sensitive strategies that integrate cardiovascular prevention into comprehensive women’s health policies, moving beyond cancer-centered approaches.

## Introduction

In 2019, the top 10 causes of death accounted for 55% of global mortality, with ischemic heart disease (IHD) remaining the leading cause, responsible for 16% of all deaths. Notably, IHD has shown the steepest rise over recent decades, increasing from just over 2 million deaths in 2000 to 8.9 million in 2019.^1^

In Latin America and the Caribbean (LAC), cardiovascular disease (CVD) is the leading cause of death, with cause-specific mortality rates of 184 per 100,000 among adult men and 108 per 100,000 among adult women – substantially higher than the averages reported in Organization for Economic Co-operation and Development (OECD) countries (104 and 53 per 100,000, respectively).^2^ Moreover, in younger women, mortality from acute myocardial infarction has been reported as higher than in men across several regions.^3-6^ Since then, new epidemiological data have emerged, and the COVID-19 pandemic has substantially reshaped mortality trends worldwide, including in LAC, where it temporarily surpassed CVD as the leading cause of death. These shifts underscore the need for updated analyses and new review papers to reassess the evolving burden of CVD, particularly in women.

Although CVDs are the leading cause of mortality among women in LAC, awareness of cardiovascular disease in women remains low across the region.^7-12^ Some studies show that LAC women are more likely than men to present with increased adiposity indicators and with three or more cardiovascular risk factors.^13^ Overall, most CVD cases and premature deaths in LAC could be prevented through effective control of metabolic risk factors and tobacco use, which remain the most common and powerful drivers of both outcomes in the region.^14,15^

Women continue to be underrepresented in key sectors such as the economy, education, and scientific research, perpetuating structural disparities. Gender equality is not only a fundamental human right but also a critical driver of global health, equity, and sustainability. Recent health crises, including the COVID-19 pandemic, along with the ongoing effects of climate change, have further influenced cardiovascular mortality and the prevalence of risk factors, amplifying existing gender gaps.^16^ Addressing these challenges requires the generation of reliable, up-to-date data capable of guiding health policies aligned with the Sustainable Development Goals – particularly SDG 3: Good Health and Well-Being – by monitoring risk factors and their impact on communities.^12,17,18^

To have an updated overview and understand the mechanisms that contribute to the increase in CVD in women, the International Atomic Energy Agency (IAEA) initiated a regional project aiming to improve awareness of CVD by identifying gaps in education and training. The IAEA provides technical cooperation support to 31 countries in LAC. The program helps Member States to address key developmental priorities in areas such as health, environment, and industrial applications.

This paper aims to describe the current situation of the CVD in the region of the Americas, as a tipping point for the implementation of initiatives, as part of the IAEA-supported ARCAL 6086 project “Integrating Nuclear Medicine Techniques in a multimodality approach in cardiology for early diagnosis and risk stratification of cardiovascular disease in Latin American women”. This study provides a regionally grounded, and sex-specific analysis of CVD in women in LAC. By integrating cross-country mortality data, premature CVD risk (ages 30–70), traditional cardiometabolic factors, female-specific life-course determinants, and the impact of COVID-19 within a unified framework, we aim to move beyond descriptive burden estimates and offer a context-sensitive, policy-oriented perspective tailored to the realities of LAC health systems.

## Methods

This study was designed as a descriptive ecological analysis based exclusively on secondary data retrieved from the Pan American Health Organization (PAHO) ENLACE data portal for noncommunicable diseases and mental health.^18^ The objective was to characterize the burden of CVD and associated risk factors in women across countries in LAC, within the broader region of the Americas.

We analyzed the most recent available data from PAHO, including mortality rates and disability-adjusted life years (DALYs) for 2021. In addition, we examined the prevalence of major atherosclerotic risk factors using 2022 data for diabetes, insufficient physical activity, overweight, and obesity; 2019 data for hypertension prevalence; and 2018 data for tobacco smoking rates and mean total cholesterol levels.

The study reports standardized epidemiological indicators as provided by PAHO, including incidence, prevalence, mortality rates, and DALYs. Data were examined for all age groups and stratified by sex (females, males, and both sexes combined). Emphasis was placed on female-specific mortality patterns and premature CVD mortality (ages 30–70 years), when available.

The 36 countries included in the analysis were: Antigua and Barbuda, Argentina, Bahamas, Barbados, Belize, Bolivia (Plurinational State of), Brazil, Canada, Chile, Colombia, Costa Rica, Cuba, Dominica, Dominican Republic, Ecuador, El Salvador, Grenada, Guatemala, Guyana, Haiti, Honduras, Jamaica, Mexico, Nicaragua, Panama, Paraguay, Peru, Puerto Rico, Saint Kitts and Nevis, Saint Lucia, Saint Vincent and the Grenadines, Suriname, Trinidad and Tobago, United States of America, Uruguay, and Venezuela (Bolivarian Republic of). Although the United States and Canada are not LAC countries, they were included for comparative purposes, consistent with PAHO regional classification of the Americas.

ENLACE functions as a comprehensive regional repository that integrates primary data from national vital statistics systems officially reported by Member States, population-based health surveys, and harmonized global health estimates from the World Health Organization (WHO) and the Global Burden of Disease (GBD) study. These integration procedures are designed by PAHO to enhance data standardization and cross-country comparability.

The analysis focused on describing and comparing standardized epidemiological indicators across countries and sexes. All results are presented as descriptive estimates derived from PAHO-reported data, and interpretations are limited to population-level patterns without implying causal associations.

This study used publicly available, aggregated secondary data from the PAHO ENLACE platform; therefore, ethics committee approval and informed consent were not required. All data are accessible through the PAHO portal, and no primary datasets or analytical code were generated.

## Results

### Global burden of cardiovascular disease in LAC countries

Cardiovascular diseases – principally IHD and cerebrovascular disease, mainly stroke – remain leading causes of mortality and major contributors to DALYs. In 2021, COVID-19 was the foremost cause of disease burden in the Region of the Americas for both sexes.^19^

According to the latest PAHO estimates for 2021, the leading causes of death in the Americas were: COVID-19 (176.7 deaths per 100,000 population), IHD (114.0 per 100,000), and stroke (48.4 per 100,000), followed by Alzheimer’s disease and other dementias (38.1 per 100,000) and chronic obstructive pulmonary disease (COPD) (34.6 per 100,000). When stratified by sex, the second and third leading causes of mortality remained IHD and stroke; however, the fourth cause differed: in men, interpersonal violence; in women, Alzheimer’s disease (see Figure 1).^18^

**Figure 1:** – Leading causes of death and health loss in the Americas, 2021 (both sexes, male, and female). Modified from World Health Organization.47

Premature mortality from CVDs – measured as the unconditional probability of dying between the exact ages of 30 and 69 years – is one of the indicators monitored by the World Health Organization. The higher this probability, the greater the impact of CVDs on mortality in a given country. Disaggregating these data by sex helps illuminate disparities in the CVD burden between men and women.

Figure 2 shows the probability of dying from CVDs between ages 30 and 69, stratified by sex. The country with the lowest premature CVD mortality among women in the region is Canada (1.7%). This probability is more than 12 times higher in Haiti (21.9%). Among men, Peru (4.4%) and Guatemala (4.4%) had the lowest premature CVD mortality. At the opposite end, the highest probabilities were also observed in Haiti (20.1%) and Guyana (17.7%).

**Figure 2:** – Probability of dying from cardiovascular diseases between exact ages 30 and 69 divided by sex (expressed in percentage). Modified from World Health Organization.48

The probability of dying from CVDs between ages 30 and 69 is higher in women only in Haiti (1.8 percentage points higher in women than in men). In all other countries in the Americas, premature CVD mortality is higher in men than in women. Suriname and Guyana showed the largest sex differences in this risk: 6.7 percentage points higher among men in Venezuela and 6.2 percentage points higher in the Dominican Republic.

### Atherosclerotic risk factors

Cardiovascular risk factors for women included in this clinical review are the ones traditionally known, such as: high blood pressure, diabetes mellitus, smoking habit, obesity, sedentary lifestyle. Additionally, sex-specific factors have been found for the feminine condition tied to their background.^20-26^ The following analysis evaluates which countries have a higher prevalence and the potential underlying causes. All the countries in the Americas Region were included in the analysis to facilitate comparison.

#### Cholesterol levels

Age-standardized mean total cholesterol across countries of the Americas in 2018 ranged from 164 mg/dL in Barbados to 185 mg/dL in Uruguay for men, and from approximately 173 mg/dL in Barbados to over 192 mg/dL in Guyana for women (Figure 3a). In almost all countries, women showed higher cholesterol levels than men, except for Chile.

**Figure 3:** – Main cardiovascular risk factors in countries of the America: Mean total cholesterol level (a); prevalence of diabetes in adults (b); Current tobacco smoking rates among people aged 15 years and older (age-standardized estimate) (c); prevalence of hypertension among adults aged 30-69 years (age-standardized estimate) (d). Modified from Pan American Health Organization49,50 and World Health Organization.51

#### Prevalence of diabetes in adults

The prevalence of diabetes in adults (fasting blood glucose ≥126 mg/dL, HbA1c ≥6.5% or currently taking medication) among adults aged 18 years and older is shown in Figure 3b. Prevalence varied widely across countries, ranging from about 7.7% in Canada to more than 33.7% in Trinidad and Tobago for women. Among men, the lowest prevalence was observed in Panama (7.8%) and the highest in Puerto Rico (27.2%). On average, prevalence in LAC countries was notably high – affecting one in five women (20%) and nearly one in six men (16%) – as illustrated in the Central Illustration.

The prevalence of diabetes in adults was generally higher among women than men across most countries, with exceptions in the United States, Canada, Venezuela, Bolivia, and Guatemala. The largest sex gaps were observed in Dominica, Jamaica, and Barbados, where women had a markedly higher prevalence than men.

#### Smoking

In 2018, the prevalence of smoking was consistently higher among men than women across all countries of the Americas. Among women, prevalence was below 20% in nearly all countries, except in Chile, where 32.7% of women reported smoking (Figure 3c). The countries with the highest female smoking prevalence were Chile (32.7%), the United States (17.3%), Argentina (16.6%), and Uruguay (16.0%). At the other end, the lowest prevalence among women was observed in Barbados (2%), Panama (2.1%), and El Salvador (2.2%).

Among men, smoking prevalence was substantially higher, surpassing 30% in Chile and Cuba. The largest sex gaps were seen in Cuba, Guyana, and El Salvador, with Cuba showing the greatest disparity—smoking was 25.2 percentage points more prevalent in men. In contrast, the Dominican Republic displayed the narrowest gap between sexes, with only a 3.8 percentage point difference.

#### High blood pressure (HBP)

A heterogeneous prevalence of hypertension was identified among adults aged 30–79 years (Figure 3d). Hypertension was defined as a systolic blood pressure ≥ 130 mmHg, a diastolic blood pressure ≥80 mmHg, or the self-reported use of antihypertensive medication.

The countries with the highest quintile of HBP were Paraguay (56.4%), Dominican Republic (49.1%), Dominica (47.7%), Argentina (47.5%), Grenada (46.6%), Jamaica (46.3%), Saint Kitts and Nevis (45.1%) and Brazil (45.0%). In comparison, Guatemala (32.2%), Mexico (32.1%), USA (31.6%), Colombia (31.0%), Bolivia (28.3%), Ecuador (27.2%), Canada (22.1%) and Peru (20.7%) were in the lowest quintile. Interestingly, the hypertension prevalence among sex were relatively lower among women in most countries. Argentina and Paraguay showed the higher differences between sexes concerning prevalence of HBP: 12.8pp higher among men in Argentina (54.0% vs 41.2%) and 10.7pp higher among men in Paraguay (61.6% vs 50.9%). On the opposite direction, Haiti demonstrated the highest prevalence of HBP among women in comparison to men, with 10.2pp higher in women. (37.6% vs 47.8%). The country with the highest prevalence of HBP among women was Paraguay (50.9%).

#### Insufficient physical activity

Unlike arterial hypertension, insufficient physical activity is more prevalent among women than men across LAC. The proportion of sedentary women is highly heterogeneous across the region (Figure 4a). Countries in the highest quintile of physical inactivity among women were Cuba (72%), Panama (66.9%), Costa Rica (57.8%), Suriname (55.1%), and Trinidad and Tobago (54.2%). In contrast, the lowest prevalence was observed in Saint Lucia (24.0%), Bolivia (26.6%), Ecuador (27.8%), Mexico (30.5%), and Haiti (35.8%).

**Figure 4:** – Prevalence of insufficient physical activity among adults 18+ years (age-standardized estimate) (a) and of overweight and obesity - body mass index ≥ 25 kg/m2 (b) in countries of the Americas. Modified from World Health Organization.52

A sedentary lifestyle was consistently more common among women. The largest sex gaps were seen in Cuba, Guyana, and Bahamas, each showing more than 20 percentage points higher prevalence of inactivity among women compared to men. On average, in LAC countries, 44% of women and 31% of men reported insufficient physical activity (Central Illustration).

#### Overweight and obesity

The prevalence of overweight and obesity (body mass index ≥25 Kg/m^2^) in countries of the Americas by sex is shown in Figure 4b. Among women, prevalence was very high, ranging from 38.4% in Haiti to 81.9% in Saint Kitts and Nevis. Among men, the lowest prevalence was observed in Haiti (21.0%), while the highest was in the Chile (78.5%). Overall, overweight and obesity were more common in women than in men in nearly all countries, with exceptions in Canada, the United States, Argentina and Uruguay.

The largest female–male differences were found in Dominica, Jamaica, Saint Vicent and the Grenadines and Saint Lucia, where prevalence among women exceeded that of men by more than 25 percentage points. On average, in LAC countries, 70% of women and 59% of men were overweight or obese (Central Illustration).

## Discussion

This study provides a regionally grounded analysis of CVD burden in LAC women highlighting that despite the exceptional impact of the COVID-19 pandemic, CVD remains the leading cause of death in the region. Our findings, derived from ENLACE database, demonstrate a concerning prevalence of traditional cardiometabolic risk factors that disproportionately affect LAC women compared to men. While these data underscore the magnitude of the problem at a population level, the transition from these epidemiological patterns to individual clinical risk is heavily influenced by sex-specific determinants. Biological factors and adverse pregnancy outcomes serve as powerful multipliers of risk that remain under-recognized in current regional health agendas.

In this context, it is essential to discuss how female-specific conditions interact with the traditional risk profile observed in our analysis. It is well recognized that both genetic and environmental factors can significantly modify the cardiovascular risk profile in women. Some are directly influenced by sex hormones, particularly estrogens, which exert protective cardiovascular effects during the reproductive years, while others are shaped by genetic and epigenetic mechanisms, including pregnancy-related disorders, that impact risk throughout the life course. In the context of LAC, several of these determinants have been specifically studied, highlighting the need to consider women’s cardiovascular health from a life-course perspective, spanning reproductive, climacteric, and postmenopausal phases.^27-29^

Polycystic ovary syndrome affects 4-20% of women of reproductive age. Its reported prevalence in Latin-American women is variable: it is estimated a 6.6% (95% CI: 2.3 to 10.9%) in Mexican women, and around 8.5% in Central and South America (see Central Illustration). The diagnosis is established by suspecting two of the following criteria: 1) hyperandrogenism, 2) polycystic ovaries, and c) ovarian dysfunction. It is associated with a higher risk (5-10 times) of cardiometabolic risk factors, such as obesity, metabolic syndrome, type 2 diabetes mellitus, high blood pressure and dyslipidemia, which increase the risk of CVD compared to normal population (OR 1.5, 95% CI 1-1.2); therefore, lifestyle care is important in the control of overweight, obesity and the development of diabetes mellitus.^30,31^

Pregnancy-related disorders are increasingly recognized as important sex-specific cardiovascular risk factors. Conditions such as hypertensive disorders of pregnancy (e.g., preeclampsia, eclampsia), gestational diabetes, and preterm delivery significantly increase the risk of cardiovascular morbidity and mortality both during pregnancy and later in life. In LAC, these complications are particularly concerning due to structural inequalities: many women face barriers to healthcare access, limited screening opportunities, and low awareness among physicians of their long-term cardiovascular implications. Addressing these gaps requires greater education of healthcare providers, systematic inclusion of pregnancy history in cardiovascular risk assessment, and the integration of these variables into electronic health records and national prevention guidelines. Expanding access to postpartum follow-up and targeted prevention programs in low- and middle-income countries will also be essential to reduce the long-term burden of CVD among women.^32^

The reported global prevalence of gestational diabetes varies widely, ranging from 1% to 28%, and is approximately 8.5% (95% CI: 3.9%–14.7%) in LAC (see Central Illustration). Specifically, the International Diabetes Federation reports a prevalence of 20.7% in North America and the Caribbean, 15.8% in Central and South America, and 8.1%, 10.6%, and 9.8% in Argentina, Brazil, and Chile, respectively, with even higher prevalence in Cuba (24.6%) and Mexico (13.3%).^33,34^

Women with gestational diabetes have a higher incidence of cardiovascular events in the years following childbirth. Their children also face increased risks of obesity, prediabetes, and type 2 diabetes from early childhood and throughout life (20%). Women with gestational diabetes have a substantially increased likelihood of developing type 2 diabetes and metabolic syndrome (20–70%), as well as a two-fold higher risk of major cardiovascular events in the years after delivery compared with women without the condition. Therefore, after childbirth, every woman diagnosed with gestational diabetes should receive counseling on lifestyle modification and on the long-term risks of developing these conditions, including the elevated risk of cardiovascular events more than 10 years after delivery.^33^

Hypertensive disorders of pregnancy are among the leading causes of maternal and fetal morbidity and mortality. They encompass several conditions with distinct prognoses and management approaches, including gestational hypertension, preeclampsia, eclampsia, and preeclampsia superimposed on chronic hypertension.^35^ There is an exponential increase in the risk of developing CVD after a pregnancy complicated by preeclampsia, and this risk rises with age: a 20-year-old woman with a baseline risk of 0.6% sees it increase to 1.2%, whereas a 40-year-old woman’s risk increases from 5.6% to 11.2%.^36^ The global incidence of hypertension in pregnancy rose from 16.30 million (95% CI: 13.56–19.42 million) in 1990 to 18.08 million (95% CI: 15.26–21.11 million) in 2019, representing a 10.92% increase.^37^ In 2019, incidence per 100,000 women varied substantially across LAC. As shown in the Central Illustration, rates were highest in Central and Tropical subregions (4.87, 95% CI: 4.27–5.54) and lowest in the Caribbean (0.86, 95% CI: 0.69–1.04).^37,38^ The elevated risk of CVD persists for 10 to 20 years after pregnancy, underscoring the importance of blood pressure monitoring and early lifestyle modification following a hypertensive pregnancy.^35^

Preterm birth, defined as delivery before 37 weeks of gestation, is also associated with increased cardiovascular mortality. The mechanisms linking preterm birth to CVD involve preexisting cardiovascular risk factors, systemic inflammation, and subsequent vascular dysfunction. Reported incidence varies globally, with the lowest rates (<5%) in Europe and the highest in Africa (18%). Approximately 60% of preterm births worldwide occur in sub-Saharan Africa and South Asia. LAC occupies an intermediate position, with notable variation across subregions (25–30%, see Central Illustration). It is estimated that 5.8% to 7.1% of pregnant women worldwide have experienced at least one preterm birth. Assisted reproductive technologies are also associated with higher risks of preterm and early-term birth, and their use has increased in Latin America, particularly among women with singleton pregnancies.^39^

Rheumatic heart disease causes 0.7 deaths per 100 000 inhabitants in the region of the Americas. Valvular heart disease is a major contributor to loss of physical function, quality of life and longevity in the population. Rheumatic disease remains by far the most common manifestation of primary disease worldwide and affects approximately 40.5 million people, with an incidence of 2.8 million per year, and accounted for 306,000 global deaths in 2019, at a median age of 28.7 years in low-income and middle-income countries.^40^ The epidemiology of this disorder was analyzed with a focus on geographical distribution and sex differences in the Americas.

Regarding the contribution to overall CVD mortality in the region, the countries with the highest rates among women in 2019 were: Haiti, Bolivia, and Saint Lucia. Women experienced 50.9 million DALYs each year in Brazil, Costa Rica, Nicaragua, and Saint Lucia, with lower levels in Venezuela, Colombia, Uruguay, and Chile (2.0 million DALYs each year) (see Central Illustration). Men had 9.2 million DALYs in Costa Rica, Nicaragua, Saint Lucia, Granada, and Brazil, with lower levels in Venezuela, Colombia, Chile, and Uruguay (1.4, 1.5, 2.6 and 2.7, respectively). There is a substantial within-region national and sub national variability in rheumatic heart disease DALYs, some examples of which include substantially higher in Brazil, Costa Rica and Nicaragua compared to the rest of LAC.^40-42^

Our analysis of CVD in women across LAC highlights several key reflections. First, CVD remains highly prevalent, with IHD among the leading causes of mortality in women throughout the region. Second, traditional cardiovascular risk factors show marked heterogeneity between sexes. Third, female-specific risk factors are both highly prevalent and frequently overlooked. Conditions such as teenage pregnancy, preterm birth, gestational diabetes, and hypertensive disorders of pregnancy are strongly shaped by socioeconomic determinants and remain under-recognized despite their well-established contribution to lifelong cardiovascular risk. Taken together, these findings underscore the urgent need for tailored strategies to promote cardiovascular health in women in the region, integrating biological, cultural, and social determinants.

When examining conventional risk factors, most LAC countries show higher rates of cholesterol, hyperglycemia, insufficient physical activity, and overweight/obesity among women, whereas smoking remains the only risk factor consistently higher in men. This imbalance is concerning given that women’s health programs in the region have historically emphasized cancer prevention (e.g., breast, cervical, ovarian), while dedicating less attention to CVD prevention—even though CVD is the leading cause of death in women outside of the COVID-19 pandemic years. Indeed, in 2021, COVID-19 emerged as the foremost cause of mortality in the Americas (176.7 deaths per 100,000 population), surpassing IHD (114.0/100,000) and stroke (48.4/100,000). While this surge represents an exceptional circumstance, it further complicated prevention priorities and strained healthcare systems, highlighting the vulnerability of women with pre-existing cardiometabolic risk factors.

The Global Use of Strategies to Open Occluded Coronary Arteries in Acute Coronary Syndromes (GUSTO IIb) study, published two decades ago, had already documented sex differences in cardiovascular risk factors and in the presentation of acute coronary syndromes: women were older than men and more frequently hypertensive and diabetic, with higher cholesterol levels and a more common history of angina, heart failure, and cerebrovascular disease.^20^ Similar findings have been consistently reported, reinforcing the need to adequately include women in clinical studies and to avoid extrapolating CVD data from men to women.^20^

The 2019 American Heart Association survey showed a decline in women’s awareness of CVD as their leading cause of death, dropping from 65% in 2009 to 44% in 2019. The steepest declines occurred among younger, Hispanic, and Black women, with many more incorrectly identifying cancer as the primary cause of death. These findings underscore the urgent need for renewed education and awareness campaigns tailored to women’s cardiovascular health.^2 3^ In some LAC countries, similar surveys have been conducted, revealing that fewer than 20% of respondents recognize CVD as the main cause of death.^24,25^

When disaggregated by sex, annual global deaths and DALYs among women were approximately 13% lower than among men; however, women spent about 26% more years living with disability (YLDs).^21^ Current women’s health guidelines have traditionally emphasized the prevention of breast, cervical, and ovarian cancers, while giving comparatively less attention to CVDs – the leading cause of death among women, except during the COVID-19 pandemic. Preventive care strategies often prioritize reproductive health and cancer screening, yet evidence shows that women face a disproportionately high burden of cardiometabolic risk factors, including obesity, hypertension, diabetes, and dyslipidemia, which significantly increase their lifetime risk of CVD. Despite this, screening and counseling for cardiovascular health remain underutilized compared with cancer-focused interventions. This imbalance must change, as both CVD and cancer warrant dedicated preventive strategies due to their high burden and overlapping risk factors.^22^ In alignment with the United Nations Sustainable Development Goals, the target for 2030 is to reduce premature mortality from non-communicable diseases by one-third through prevention.

Premature mortality data further underscore the uneven impact of CVD across countries. Among women, the probability of dying from CVD between ages 30 and 70 was highest in Haiti (21.9%) and Guyana (15.2%). In Haiti, hypertension stands out as the dominant risk factor (47.8%), while diabetes and smoking rates are relatively low, suggesting that deficiencies in the diagnosis, treatment, or management of hypertension may be driving the country’s elevated premature mortality. Guyana, conversely, showed some of the highest cholesterol levels among women in the region, along with above-average prevalence of other cardiometabolic risk factors, compounding the overall risk burden.

At the opposite end of the spectrum, Canada presented the lowest female premature mortality from CVD (1.7%). This favorable profile likely reflects the advantages of a high-income setting with stronger healthcare infrastructure, better access to preventive care, and more effective management of risk factors. Indeed, Canada’s prevalence of hyperglycemia, hypertension, sedentary lifestyle, and overweight/obesity were all below regional averages. Interestingly, smoking prevalence among Canadian women (10.5%) was about three times higher than in Haiti (3.4%), yet overall mortality remained substantially lower. This finding reinforces the notion that the clustering and coexistence of multiple risk factors – rather than isolated exposures – play a decisive role in driving disease burden. Understanding these interactions is crucial for designing more effective and context-specific prevention strategies in LAC.

Although our findings align with previously reported patterns – such as the predominance of IHD and the high burden of metabolic risk factors – our analysis extends prior benchmarks by focusing specifically on premature female CVD mortality (ages 30–70) and highlighting the marked cross-country heterogeneity across LAC.^27,32,43,44^ We also integrate traditional cardiometabolic factors with female-specific life-course determinants, including teenage pregnancy and hypertensive disorders of pregnancy, within a unified framework. Additionally, situating our results within the post-COVID-19 mortality context underscores how underlying cardiometabolic vulnerability amplified regional disparities, providing a more comprehensive and policy-relevant interpretation of cardiovascular risk in women.

Our findings underscore the need for coordinated, sex-specific cardiovascular strategies in LAC. Public health authorities should expand women’s health agendas beyond cancer prevention to include systematic cardiovascular risk assessment across the life course, particularly targeting hypertension control, diabetes screening, lipid management, and physical inactivity. Structured follow-up of women with adverse pregnancy outcomes – such as hypertensive disorders of pregnancy and gestational diabetes – should be incorporated into primary care pathways. Health systems must also optimize diagnostic and treatment pathways for IHD in women, ensure equitable access to guideline-directed therapies, and strengthen referral to cardiac rehabilitation.

Given the marked heterogeneity in premature female CVD mortality across countries, interventions should prioritize scalable, cost-effective measures – especially blood pressure control – while implementing measurable indicators, including sex-disaggregated screening rates, risk factor control, and premature mortality (ages 30–70), to monitor progress over time.

Key findings from the most recent 2024 and 2025 studies on heart disease in women in Latin America indicate that socioeconomic disparities, urbanization, and social determinants of health are major drivers of risk and outcomes, with rural women facing greater barriers to care and higher mortality rates.^43,45,46^ There is a critical need for tailored interventions that address country-specific variations in healthcare infrastructure, access, and political stability.

We acknowledge that the use of different reference years for distinct cardiovascular risk factors may introduce temporal heterogeneity and potentially affect direct comparability between indicators. The findings should therefore be interpreted in light of this inherent characteristic of international open-access databases.

## Conclusion

In summary, CVD remains the leading cause of death among women in LAC, driven by a complex interplay of traditional and female-specific risk factors, many of which are under-recognized and insufficiently addressed in current health agendas. The marked disparities between countries highlight the influence of socioeconomic context, healthcare access, and clustering of risk factors on outcomes. These findings underscore the urgent need for sex-specific, context-sensitive strategies to improve prevention, early detection, and management of CVD in women, as well as stronger integration of cardiovascular health into existing women’s health programs across the region.
